# Nursing Students' Perceptions on Healthcare-Associated Infection Control and Prevention Teaching and Learning Experience: Development and Validation of a Scale in Four European Countries

**DOI:** 10.3389/fpsyg.2021.701208

**Published:** 2021-10-08

**Authors:** Amaia Yurrebaso Macho, Alexander L. Ward Mayens, Eva Ma Picado Valverde, Raquel Guzmán Ordaz, Juan Antonio Juanes Méndez, Jose Luis Pérez Iglesias, José Antonio Mirón Canelo, Maria do Rosário Pinto, Alcinda Maria do Sacramento Costa Reis, Joaquim Augusto Simões, Ana Luísa Torres, Marja Silén-Lipponen, Ulla Korhonen, Leena Koponen, Mikko Myllymäki, Aleksandra Jankowiak-Bernaciak, Amelia Patrzała, Grażyna Bączyk, Anna Basa, Paulo Santos Costa, Beatriz Serambeque, Anabela Salgueiro Oliveira, João Pardal, João Manuel Garcia do Nascimento Graveto, Pedro Parreira

**Affiliations:** ^1^Salamanca University, Salamanca, Spain; ^2^IP Santarem, Santarem, Portugal; ^3^Savonia University of Applied Sciences, Health and Social Care, Kuopio, Finland; ^4^Hipolit Cegielski State University of Applied Sciences in Gniezno, Gniezno, Poland; ^5^Nursing School of Coimbra (ESEnfC), Coimbra, Portugal

**Keywords:** infections, infection control, learning, nursing, students

## Abstract

Healthcare-associated infections are one of the major concerns worldwide. This study presents the development and the validation process of the InovSafeCare scale and aimed at identifying and measuring the ecosystem variables related to healthcare-associated infection (HCAI) prevention and control practices in European nurse students. Qualitative and quantitative approaches were used to (1) elaborate an item pool related to the educational environment, the healthcare setting environment, and the attitudes, beliefs, and performance of the nursing students regarding HCAI prevention and control and (2) analyze psychometric properties of the scale using factor analysis. The validated InovSafeCare scale was applied to undergraduate nursing students of five European Higher Education Institutions. The partial least square structural equation modeling (PLS-SEM) method with SMART-PLS3 software was used. The study sample consists of 657 nursing students, who responded a self-report inventory. From the analyzed data were identified 14 factors. The InovSafeCare scale reveals good validity and reliability of the dimensions in different European countries.

## Introduction

Healthcare-associated infections (HCAIs) are a major concern worldwide, not only because it prolongs hospital stay, with the consequent increased costs for health systems, but also because of the repercussions on the health of patients (European Center for Disease Prevention Control (ECDPC), 2020; World Health Organization, 2020).

In Europe, about 4 million patients acquire an HCAI every year, of which 37,000 end up in deaths [European Center for Disease Prevention Control (ECDPC), 2020]. The ECDPC estimates that around 30% of HCAIs can be prevented with surveillance systems, and prevention and hygiene control programs (2020), meaning they become an indisputable preventive content in healthcare institutions for their different professionals, as well as academic content necessary for all healthcare students [Díaz and Cadena, 2003; Agencia Nacional de Evaluación de la Calidad y Acreditación (ANECA), 2004].

Brosio et al. (2017) study showed that nursing students demonstrate an insufficient knowledge about HCAIs (Brosio et al., 2017). Additionally, nursing students are active practitioners in healthcare institutions, being exposed to a high risk of acquiring infections due to the type of tasks they perform, the direct contact with potentially infectious patients, and the handling of blood and body fluids (Díaz and Cadena, 2003; Rodríguez et al., 2008; Rubio et al., 2008). Despite the previous preparation obtained in laboratory practice and a simulation environment, the perception of risks of infection, attitudes, and motivations toward safe practices and prevention itself through the use of personal protective equipment (PPE) are at the root of this accident rate (Herrera and Gómez, 2003; Ortiz, 2003; Organización Panamericana para la Salud, 2007; Siesto, 2017).

Nursing students are exposed to highly contaminated environments (by either contact with patients, family members, or health personnel who may carry infections), and it is estimated that they are 40% more likely to be infected than the rest of the general population (Cabezas, 2012). According to Tapias et al. (2010), it is through the knowledge of exposure mechanisms and transmission risks, and their prevention that healthcare students and workers can contribute to creating a safe working environment, hence the need to invest in more and better education in this subject, with theoretical classes, theoretical-practical, and simulated practices.

Educationally wise, the implementation of the Bologna Plan (1999) has meant a change in the educational paradigm from teacher-based learning to a student-centered model. This implies a new dynamic in pedagogical methodologies for the acquisition of theoretical knowledge and the development of theoretical-practical competences within the clinical context (Siles González, 2011).

Specifically, in the scope of HCAIs, education needs to accompany the guidelines on core components of infection prevention and control (IPC), based on the priority topics identified, among others, by World Health Organization (2020), including strategies and tools for evaluation. Being a very specific and wide-ranging scientific area, where theoretical knowledge acquired is a tool that supports clinical decision-making, strategies associated with the learning/teaching process are fundamental in this educational area, permitting students to acquire and develop competences throughout their learning pathway.

These proposals, however, always face the challenge of how education and practice translate to an actual change in HCAI safe practices. Humphreys and Richards (2011), for example, discuss how teaching differs between medical and nurse students, where nurse students show a knowledge and performance advantage (Tavolacci et al., 2008; van de Mortel et al., 2010); yet, they catch up with their performance once they advance to postgraduate education. When looking into the educational factors, these changes happen partly due to a higher focus in teaching about HCAI control and prevention, increase in practice (Hunt et al., 2005), learning from supervisors (Snow et al., 2006), awareness, and a change from traditional lecture methods (Helder et al., 2010; Lobo et al., 2010). On the other hand, finding these differences between healthcare personnel education and practices suggests that reforming or improving the quality curriculums would lead to improved HCAI control and prevention practices.

Professional knowledge in nursing, as stipulated by the degree, is also characterized by being practice-oriented. Approximately 50% of the training must be developed in a clinical context, oriented toward learning in a team and in direct contact with patients and the community to organize, provide, and evaluate the care required from the knowledge, capacity, and skills acquired by the student (Zabalza Beraza, 2011; Rodríguez-García, 2019). The contents to be taught in the practices are theoretical and practical and must be developed by both academic and clinical tutors, adapting the contents and the learning process to each context (Zabalza Beraza, 2011). Clinical tutors or supervisors have a relevant role in the teaching and learning process by generating an adequate learning environment, in which other professionals from the care center also participate without educational responsibilities, influencing, and sharing habits, customs, and working conditions of all kinds (Rodríguez-García, 2019).

The introduction of clinical practices aims to teach students of the practical skills necessary to provide competent care, as the link between theory and practice facilitates the assimilation of knowledge and the development of professional skills. Practices must still follow safety standards, that is, based on interventions aimed at avoiding or minimizing harm from patient care (on Joint Commission on Accreditation of Healthcare Organization, 2015). By clinical practice, we understand the care function of the nursing profession, including all the functions and activities that it carries out to respond to the demands of health and illness. Safe HCAI practices should be based on adequate epidemiological surveillance systems that include multifaceted actions that promote safe professional practice by multidisciplinary teams (Dignath et al., 2008; WHO, 2018).

In addition to the educational organization itself (content, planning, direction, and control of the process), learning strategies can be affected by the unique situation of each student, both at the individual level (cognitive, motivational, and behaviors of the subject) and in the environment or learning context (patients, peers, other professionals, teachers, health center structure, and practices) (Quinn and McGrath, 1985; Stoner and Wankel, 1989; Gargallo, 2006). In fact, a study with nurse students showed that the best predictor of hand hygiene compliance was having their mentors practice it as well (Snow et al., 2006). The student intentionally and consciously analyzes the characteristics of the task that is demanded, the resources available for its execution, and the limitations found both on a personal level and in the learning context (Dignath and Büttner, 2008; Dignath et al., 2008; Sitzmann and Ely, 2011). Preventive practice, in short, is not going to depend exclusively on the knowledge or the availability of prevention resources. The desire of the student to put them into practice also weights in this process (Virú-Loza, 2012), a performance on which other variables can mediate besides the cognitive ones, which will have to do with his/her style of behavior, his/her personality traits, the type of affection and emotions he/she experiences at the time, and the motivations that lead him/her to execute the task.

We sum all these broad factors as a foundation to propose an HCAI prevention and control ecological system, taking into account the ecosystem in which the students develop competencies, and use this term to conceptualize what the environment entails for a nurse student. An ecosystem can be defined in its simplest form as the interactive system between organisms and an environment. The term, although rooted in biology, has also been used for social applications, where a human ecosystem is the interactional environment of a social system (individuals, groups, and cultures), with a resource system (Brush, 2014). The human ecosystem has been used in many different domains, including business, education, cybernetics, media, among others, and also in healthcare with the Workforce Ecosystem Model.

More specifically, it comprises of staffing (competence, education, and the volume of work), workflow design (on the job activities), personal/social factors (stress, job satisfaction, and professionalism), physical environment (lights, aesthetics, and sound), and organizational factors (use of teams, division of roles, and shared beliefs) (Hickam et al., 2003).

This said, using this background, we suggest the student ecosystem for HCAI control and prevention practices should involve three key agents that work as a collaborative link to engage in safe-care learning and performance: the educational and work environment, culture, and beliefs; and the student as practitioner and agent of change. We propose these are the root foundations in an ecosystem focused on student learning and development, which guides to acquire competences and values while facilitates a feasible environment to put in practice prevention and control safe practices. This proposal has a different view than the Workforce Ecosystem in a way that allows to have a central focus in the student. Consequently, it allows us to offer a unique view of the nursing environment and also narrow the scope for a more reasonable and answerable questionnaire length. With these goals in mind, we proceed to the development process of the instrument by trying to answer the question: What factors within this framework comprise the student HCAI control and prevention ecosystem?

To explore the perceptions of nursing students regarding their teaching and learning experience in the field of HCAIs prevention and control, and their attitudes and perceived performance, a European Consortium developed the InovSafeCare Questionnaire. This scale will offer the opportunity to explore and understand the current state of HCAIs prevention and control from the perspective of the educational environment, the healthcare organizations environment, and the attitudes, beliefs, and performance of the nursing students. We believe this scale serves a 2-fold purpose: to provide an empirical measurement of HCAIs-related factors within nursing students in Europe, and the potential to analyze how these factors interact to provide more meaningful and efficient proposals to improve safe practices in nurse students. Not having a previous measuring tool to focus on the HCAI of European student, we believe that the InovSafeCare scale can offer a meaningful contribution to start a theoretical framework that can help future improvement designs for safe practices.

## Materials and Methods

### Development Overview of the Questionnaire

**Figure 1:**
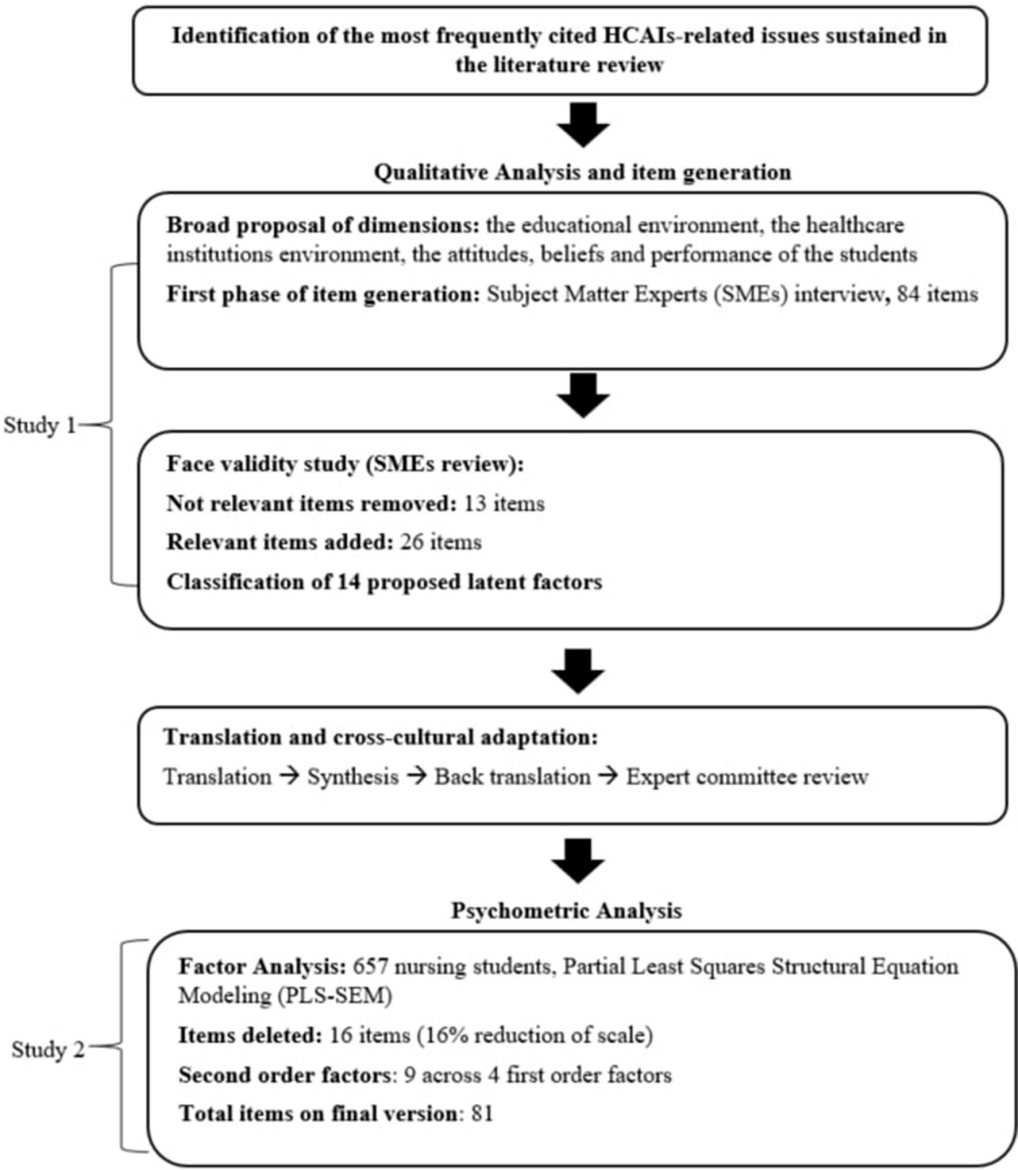
Overview of the InovSafeCare scale development process.

The development process of the InovSafeCare scale follows a mixed approach of qualitative and quantitative methods to determine the set of items, underlying the dimensions behind the object of the study, and to test the validity of the items using statistics. This process was divided into two identified studies, where Study 1 follows a qualitative approach to generate the items, while Study 2 analyzes the psychometric properties of the questionnaire through a quantitative approach. Figure 1 demonstrates an overview of the scale development process.

### Study 1: Development of Items and Their Internal Validation

This first study aimed to find the foundation of the HCAI prevention and control content-related ecosystem, develop a preliminary set of items, and adjust them using a content validity study with a panel of experts.

#### Item Generation Approach

For the development of the items, the theoretical review was previously laid out and a Content Validity Study was used to generate a set of preliminary items to be responded to in Likert-scoring format. Given the inductive approach to the item generation process, it was determined that to reach the objective with this questionnaire, the following topics must be addressed within it: the educational environment, the healthcare institutions environment, the attitudes and beliefs of the students, and the self-reported performance.

#### Content Validity Analysis

To ensure the highest quality and the well-grounded on real-life practicality of the items, the item pool development was subjected to a content validity procedure, where items were generated and submitted to a panel review process by subject matter experts (SMEs) on nursing and HCAI control and prevention practices from each of the participating institutions in the study: The Health School of the Polytechnic Institute of Santarém and the Nursing School of Coimbra from Portugal, the Hipolit Cegielski State University of Applied Sciences in Gniezno from Poland, the Savonia University of Applied Sciences from Finland, and the University of Salamanca from Spain. Each institution had at least two members in its expert review panel.

From a qualitative perspective, items can be derived by discussion according to the Standards of Educational and Psychological Testing by American Psychological Association (1985). In our case, this was facilitated by people who have direct field expertise, related to real-life experiences, and can establish indicators that are critical to our objectives. Our SME panel consisted of nurse supervisors, incumbents, and professors to guide us into what comprises the critical aspect of HCAI prevention and control within the framework of Education, Behavior, and Performance. Critical aspects were transformed into statements or questions that were further refined into an item structure and later discussed and modified until they match what SMEs considered relevant content to measure. Because we want to be as accurate as possible about the direct relevance of items and dimensions to the actual HCAI prevention and control practices, item generation benefits from discussion with SMEs in order to remain practical.

The item generation and revision phase lasted 2 months, which included virtual meetings among the panel members to discuss their feedback on each item on grammar, redundancy, latent factor correspondence, context correction, and relevance to HCAI. This further led to refining the items, ensuring beforehand a coherent topic and context for each of the latent factors, and making sure they were specifically applicable to the nursing students and safe-care topics.

#### Cultural Adaptation of the Scale

As the scale aims to encompass the European nurse framework, we worked along our consortium to have the study use a diverse sample of the European population. To make sure that the items were equally applicable and understood by the participating countries, we submitted the questionnaire to a cultural adaptation procedure. Following the methodology proposed by Beaton et al. (2020), the items were created in English, and each of the respective countries participating in the study utilized, both, a translator with knowledge about the topic, and one without, to translate it. Both translators proceeded to convert each item, as well as instructions, to the language and wording that best fits their respective culture, and then delivered to the review panel committee of each respective institution to certify faithfulness to the original English version, as well as to the local culture. From these two translated versions, a synthesized third version was created, which was composed of a selection of the better fit of each of the items from the two versions, which was then evaluated by the experts of each institution, and then back-translated into English and sent to evaluate whether translating the questionnaire from the non-English version would still retain the same meaning as the original version.

### Study 2: Statistical and Psychometric Analysis

#### Factor Analysis

The scale utilizes, both, formative and reflective factors. A formative factor is defined as when a packet of low-correlated indicators forms the latent factor. As our example, a factor about how dynamical is an institution in teaching about safe practices may have one item about the development of workshops and another about the use of visuals in classrooms for teaching students about HCAI. In this case, the increase in or presence of one does not need to relate to the change in the other. In other words, the sum of the indicators forms the construct. On the other hand, a reflective factor is when the latent construct manifests itself in a packet of correlated indicators. For example, one of our constructs has one item about how interested is the student to continue pursuing a nurse career and another about whether being a nurse will fulfill its professional expectations. The change in these items would be expected to correlate with one another, as the change in both is caused by the latent factor, *Career Motivation*.

Because the scale includes both types, covariance-based methods (or CBM) are not adequate to analyze the factor structure, as formative factors are not expected to correlate between the items (Hair et al., 2014). For this, we decided to use the partial least square (PLS) method using structural equation modeling (SEM) to test whether our proposed factors retain their structure (PLS-SEM). We used the software SMART-PLS3 to reach this objective, as its algorithm is capable to compute scores for formative (i.e., weights) and reflective (i.e., loadings) factors simultaneously in a single model.

While this method offers the advantage of validating formative constructs, it comes at the consequence of being unable to compute model fit scores traditionally found in CBM, as such, we are not including any of the CBM model fit scores on this report (i.e., chi-square test, comparative fit index, Tucker–Lewis Index, and root mean square error of approximation). Instead, current research in PLS-SEM suggests that model fit scores for these type of statistics are still at early stages (Hair et al., 2018), and thus should be cautious when reporting any. We chose to limit this to exclusively report the standardized root mean square residual (SRMR), which can be used to avoid model misspecification (Henseler et al., 2014). Values <0.1 or 0.08 are considered a good fit.

#### Second-Order Constructs

We explored second-order constructs in the case that the weights or loadings showed a pattern that would allow us to further group them within the factor and, if when revising the items, they made sense as second-order (Becker et al., 2012; Hair et al., 2018). For example, if a factor of seven items had three that showed a specific pattern (i.e., weights and/or conflicting *p*-values relative to the rest), instead of deleting them, we looked if they had some contextual relationship that may explain the results. If they did, we tried to make the second-order, and if the second-order had significant weight to the second and parent factor, they were retained as second-order. Specifically, we used the repeated indicator approach, which is the well-used method for estimating higher-order constructs in PLS, where the second-order construct is directly measured by reusing the items in the first-order factor (Wilson and Henseler, 2007).

As our example, a factor about prevention culture in health center showed two of its items show weight and *p*-value pattern different relative to the rest. When looking at the item text, these two items were about giving feedback to the student about safe practices during their placements, while the others were about personnel following and promoting compliance in the health center. By their nature, these two items correspond to the latent factor (*Health Institution HCAI prevention culture*), thus, we created second-order constructs, dividing this set of themes (*Feedback* and *Compliance*). Both had similar, and significant weights to the parent factor, thus, we retained the items by allowing second-order constructs.

#### Construct Reliability and Validity

Reflective constructs are expected to correlate, and we used loadings, instead of weights, and validity measures for internal consistency generally expected of these types of constructs. Composite reliability (CR) must be above 0.7, and the average variance extracted (AVE) should be above 0.5 (Hair et al., 2010). Below, but close to 0.5, is also permissible, since “AVE is a more conservative measure than CR. Based on CR alone, the researcher may conclude that the convergent validity of the construct is adequate, even though more than 50% of the variance is due to error” (Malhotra and Dash, 2011, p. 702). Formative constructs, on the other hand, are expected to have similar and statistically significant weights to the latent factor and low collinearity. We took a more liberal approach on *p*-values on formative factors and proceeded to leave the item if the exceeded threshold was not marginal. This is because the tradeoff of statistical confidence with values close to the threshold makes sense when the contribution of an item is unique, such as those found in these types of constructs (Cenfetelli and Bassellier, 2009). We used the variance inflation factor (VIF) to identify any concern in collinearity, with a threshold of anything above 5 a concern, and inadmissible above 10.

## Results

### Study 1: Content Validity Results

After interviewing and discussing with SMEs, it was agreed that the items should cover: (1) knowledge on HCAI prevention and control; (2) the active mission in educational and clinical settings to promote, teach, and reinforce safe practices; (3) how the student views infection control and prevention practices; (4) self-efficacy for safe practices; (5) identification with the nurse role; (6) perceived fatigue; (7) degree of distraction during clinical placements by the environment; and (8) self-reported safe practices. Using these topics as guidance, a total of 84 items were developed for the panel review process.

Panel review feedback brought indications that there were missing essential indicators, less-relevant items present, and vocabulary improvements required, which prompted us to review the item pool. One of the initial constructs aimed to indicate the degree of knowledge of specific bacteria related to HCAI, where the judges pointed out that there is higher relevance in whether prevention protocols and procedures were covered as topics in higher education, arguing that these were more relevant to HCAI control and prevention. In this case, we decided to fully replace the construct (7 items) and develop a set of items related to HCAI content covered in higher education formation (12 items). An example of this new construct is to indicate whether “Safe preparation and administration of intravenous medications” is addressed in the educational institution, as opposed to indicating the degree of knowledge in “Clostridium difficile” by the student, which was the type of item found in the originally proposed construct.

We also modified vocabulary when indicated, and whether there was any relevant content that was missing in the original pool. Specifically, the panel indicated that there was a necessity to measure whether specialized infection-trained link nurse is involved in clinical rotations of students, and an adequate student to teacher ratio in laboratories, which were further generated for the scale as a single item. Additionally, it was indicated that a specific dimension measuring the motives behind following safe practices should be included, as it would allow to gauge how transcendent the well-being of patients is for practitioners. To this, a new construct measuring intrinsic and extrinsic motives for following HCAI control and prevention was developed to fill this requirement with 11 new items, using as guidance Self-Determination Theory (Ryan and Deci, 2000).

Lastly, further discussion with the committee for item-factor correspondence and nomenclature led to propose a fixed set of 14 latent factors through 97 items, which are the following: Higher Education Institution HCAI Theoretical Content (HETheory, 12 items), Higher Education Institution Approach to teach HCAI (HEApproach, 5 items), Higher Education Institution HCAI practice-related approach/resources (HEPractice, 5 items), Health Institution HCAI prevention practices (HIPractice, 7 items), Health Institution HCAI prevention culture (HICulture, 7 items), Personal HCAI Attitudes (PAttitude, 6 items), Personal objection to HCAI prevention and control measures (PObject, 5 items), Self-Efficacy on HCAI prevention and control (SelfEff, 4 items), Career Motivation (CMotivation, 4 items), Fatigue (Fatigue, 6 items), Distraction (Distract, 7 items), Intrinsic motives to comply with HCAI control and prevention measures (IMotive, 7 items), Extrinsic motives to comply with HCAI control and prevention measures (EMotive, 4 items), Self-reported HCAI practices (SRPract, 6 items). Table 1 presents the dimensions in more detail, while Table 2 shows essential modifications done during the item generation phase.

**Table 1:** InovSafeCare dimensions.

**Code**	**Name**	**Measures**
HETheory	Higher Education Institution HCAI Theoretical Content	Level of agreement in which specific HCAI-related topics are addressed in the institution
HEApproach	Higher Education Institution Approach to teaching HCAI	Level of agreement in which multiple approaches to teaching about HCAI-related topics are used in the institution
HEPractice	Higher Education Institution HCAI practice-related approach/resources	Level of agreement in which how practice-based approaches are used to address HCAI-related topics in the institution
HIPractice	Health Institution HCAI prevention practices	Degree to which HCAI control and prevention safe practices are present in the health center where the student is doing its clinical placement
HICulture	Health Institution HCAI prevention culture	Degree to which HCAI safe practices are promoted as values within the health center where the student realizes its clinical placement
PAttitude	Personal HCAI attitudes	Degree of agreement in personal attitudes toward HCAI prevention and control practices
PObject	Personal objection to HCAI prevention and control measures	Degree to which the person feels discomfort or objection toward specificities of HCAI control and prevention practices
SelfEff	Self-Efficacy on HCAI prevention and control	Degree of confidence the student perceives in its ability to execute HCAI safe practices, even under unexpected or stressful conditions.
CMotive	Career motivation	Degree of agreement in how the student feels with their current professional path as a nurse
Fatigue	Fatigue	Degree to which the student has been experiencing events or phenomena related to exhaustion or fatigue.
Distract	Distract	Degree to which certain factors may cause distraction or loss of focus in tasks during clinical placements
IMotive	Intrinsic motives to comply with HCAI control and prevention measures	Degree to which the person complies with HCAI prevention and control practices because of internal motives
EMotive	Extrinsic motives to comply with HCAI control and prevention measures	Degree to which the person complies with HCAI prevention and control practices because of external motives
SRPract	Self-reported HCAI practices	Measures the degree of agreement to which the student reports an HCAI-related breach during their clinical placements

**Table 2:** Description and justification of original item modifications during their generation phase.

**Item**	**Old text**	**Action**	**New text**	**Justification**
HETheory_A	CJD (Creutzfeldt-Jakob Disease)	Deleted	–	The degree of knowledge on these were not considered as essential for HCAI control and prevention. Items were changed to those related to education about infection control.
HETheory_B	MRSA (Methicillin-resistant Staphylococcus aureus)	Deleted	–	
HETheory_C	Antimicrobial resistance	Deleted	–	
HETheory_D	Staphylococcus aureus	Deleted	–	
HETheory_E	Herpes Simplex	Deleted	–	
HETheory_F	Bordetella pertussis	Deleted	–	
HETheory_G	Clostridium difficile	Deleted	–	
HETheory_1	–	Added	Chain of infection	Were considered more relevant and essential for HCAI control and prevention education
HETheory_2	–	Added	Individual assessment of the risk of infection during patient admission and patient placement/isolation	
HETheory_3	–	Added	Hand hygiene	
HETheory_4	–	Added	Respiratory tract infection	
HETheory_5	–	Added	Use of personal protective equipment	
HETheory_6	–	Added	Decontamination of medical/clinical devices and equipment	
HETheory_7	–	Added	Decontamination of environmental surfaces in hospitals.	
HETheory_8	–	Added	Safe use of protective clothing	
HETheory_9	–	Added	Proper clinical waste management.	
HETheory_10	–	Added	Safe preparation and administration of intravenous medications.	
HETheory_11	–	Added	Prevention of exposure to microbial agents in the workplace	
HETheory_12	–	Added	Additional precautions for specific transmission routes	
HIPract_A	There are clocks on the walls that allow us to see the time	Deleted	–	Not considered relevant
HIPract_3	–	Added	Specialized/infection control link nurses are involved in my clinical rotations	Essential and was mising
HIPract_7	Preventive means for infection are within my accessibility (equipment, protocols, etc.)	Modified	Personal protective equipment is available in key areas (e.g., outside the isolation rooms)	Wording was poor, and “key areas” was more readibly understandable
PAttitude_1	I treat all cases in my health center as risk factor of infection	Modified	There is a potential risk of HCAI in all moments of patient care delivery	“All cases” does not encompass the true risk, which is in all moments
PAttitude_2	As a student, it falls on me the complete responsibility to prevent and control infection spread	Modified	As a student, I feel responsible for preventing and controlling the spread of infections	“Complete” is not correct, as they also work in teams
PObject_A	I believe to be an exaggeration if they are always supervising hygiene measures in general	Deleted	–	Considered redundant
Distract_3	Having a conversation with the patients	Modified	A significant number of requests from patients and/or family members	A more and relevant distractor from patients is their multiple requests from themselves and family members around the area
Imotive_1	–	Added	My main drive is patient health	Nurse student's motives behind their HCAI control and prevention practices was considered an essential component behind compliance and safe practices
Imotive_2	–	Added	I understand its implications	
Imotive_3	–	Added	I aim for quality care	
Imotive_5	–	Added	I am concerned about my own safety	
Imotive_6	–	Added	I want to implement prevention measures	
Imotive_7	–	Added	I am driven by professional ethics	
Emotive_1	–	Added	I will get better grades	
Emotive_2	–	Added	Other team members would think better of me	
Emotive_3	–	Added	Other team members also follow them	
Emotive_4	–	Added	I will meet the expectations of my supervisor	

*Item names that have a letter instead of a number were original drafts that were discarded before the final version for statistical analysis was established*.

#### Sample

Once the final item pool was approved for testing, the questionnaire was applied in 2019 after approval from the Ethics Committees (Health Sciences Research Unit: Nursing (UICISA: E) of the Nursing School of Coimbra—reference 635/11-2019) to 657 nursing students from the Health School of the Polytechnic Institute of Santarém (ESS-IPS) (107), the Nursing School of Coimbra (ESEnfC) (214), the Hipolit Cegielski State University of Applied Sciences in Gniezno (PWSZ Gniezno) (119), the Savonia University of Applied Sciences (SUAS) (58), and the University of Salamanca (USAL) (158).

Gender-wise, 83 males and 573 females participated, ranging from 18 to 55 years of age, with a mean of 22.5 years.

### Study 2: Factor Analysis Results

The factor analysis of a 14-factor conceptualization of 97 items yielded low scores and high *p*-values in some of the items within the factors HETheory, HEPractice, HIPractice, HICulture, PAttitude, and Distract, with a SRMR above 0.1. More specifically, as outer weights are the measurable indicator of the item-factor relationship, this means they do not properly fit into their assigned latent factor. Regarding reflective factors, there were no cross or outer-loading concerns, however, one item showed a low beta with a high *p*-value in IMotive. These items (14) were marked for deletion. The reason these items were removed from the item pool is that they also did not adequately adjust to second-order factors either (e.g., regardless of first or second-order, they yielded low betas and high *p*-values). Table 3 presents the item-factor weights (formative) and loadings (reflective).

**Table 3:** Weights and significance.

**Interaction**	**Outer weights**		**Outer loadings**	
	**β**	***p***	**β**	***p***
HETheory_11 -> HETheory	0.358	<0.001	–	–
HETheory_12 -> HETheory	0.242	0.005	–	–
HETheory_3 -> HETheory	0.216	0.002	–	–
HETheory_5 -> HETheory	0.253	0.003	–	–
HETheory_6 -> HETheory	0.201	0.012	–	–
HETheory_9 -> HETheory	0.159	0.021	–	–
HEApproach_1 -> HEApproach	0.209	0.007	–	–
HEApproach_2 -> HEApproach	0.272	0.004	–	–
HEApproach_3 -> HEApproach	0.205	0.014	–	–
HEApproach_4 -> HEApproach	0.341	<0.001	–	–
HEApproach_5 -> HEApproach	0.360	<0.001	–	–
HEPractice_1 -> HEPractice	0.310	<0.001	–	–
HEPractice_1 -> Resource	0.408	<0.001	–	–
HEPractice_2 -> HEPractice	0.191	0.001	–	–
HEPractice_2 -> Laboratory	0.460	<0.001	–	–
HEPractice_3 -> HEPractice	0.297	<0.001	–	–
HEPractice_3 -> Laboratory	0.665	<0.001	–	–
HEPractice_4 -> HEPractice	0.199	<0.001	–	–
HEPractice_4 -> Resource	0.317	<0.001	–	–
HEPractice_5 -> HEPractice	0.404	<0.001	–	–
HEPractice_5 -> Resource	0.536	<0.001	–	–
HIPractice_1 -> HIPractice	0.174	0.008	–	–
HIPractice_2 -> HIPractice	0.374	<0.001	–	–
HIPractice_3 -> HIPractice	0.249	<0.001	–	–
HIPractice_4 -> HIPractice	0.336	<0.001	–	–
HIPractice_7 -> HIPractice	0.323	<0.001	–	–
HICulture_2 -> HICulture	0.257	<0.001	–	–
HICulture_2 -> Compliance	0.323	<0.001	–	–
HICulture_4 -> HICulture	0.235	<0.001	–	–
HICulture_4 -> Feedback	0.533	<0.001	–	–
HICulture_5 -> HICulture	0.272	<0.001	–	–
HICulture_5 -> Compliance	0.480	<0.001	–	–
HICulture_6 -> HICulture	0.222	<0.001	–	–
HICulture_6 -> Feedback	0.695	<0.001	–	–
Pattitude_2 < - Pattitude	–	–	0.752	<0.001
Pattitude_4 < - Pattitude	–	–	0.680	<0.001
Pattitude_5 < - Pattitude	–	–	0.796	<0.001
Pattitude_6 < - Pattitude	–	–	0.509	<0.001
PObject_1 < - PObject	–	–	0.797	<0.001
PObject_2 < - PObject	–	–	0.768	<0.001
PObject_3 < - PObject	–	–	0.718	<0.001
PObject_4 < - PObject	–	–	0.593	<0.001
PObject_5 < - PObject	–	–	0.675	<0.001
SelfEff_1 < - SelfEff	–	–	0.707	<0.001
SelfEff_2 < - SelfEff	–	–	0.704	<0.001
SelfEff_3 < - SelfEff	–	–	0.785	<0.001
SelfEff_4 < - SelfEff	–	–	0.761	<0.001
CMotivation_1 < - CMotivation	–	–	0.874	<0.001
CMotivation_2 < - CMotivation	–	–	0.816	<0.001
CMotivation_3 < - CMotivation	–	–	0.900	<0.001
CMotivation_4 < - CMotivation	–	–	0.863	<0.001
Fatigue_1 -> Fatigue	0.209	<0.001	–	–
Fatigue_1 -> Academic Fatigue	0.535	<0.001	–	–
Fatigue_2 -> Fatigue	0.136	<0.001	–	–
Fatigue_2 -> General Fatigue	0.236	<0.001	–	–
Fatigue_3 -> Fatigue	0.418	<0.001	–	–
Fatigue_3 -> Academic Fatigue	0.722	<0.001	–	–
Fatigue_4 -> Fatigue	0.209	<0.001	–	–
Fatigue_4 -> General Fatigue	0.406	<0.001	–	–
Fatigue_5 -> Fatigue	0.119	0.058	–	–
Fatigue_5 -> General Fatigue	0.261	0.013	–	–
Fatigue_6 -> Fatigue	0.146	0.004	–	–
Fatigue_6 -> General Fatigue	0.242	0.004	–	–
Distract_1 -> Distract	0.322	<0.001	–	–
Distract_2 -> Distract	0.324	<0.001	–	–
Distract_5 -> Distract	0.280	0.001	–	–
Distract_6 -> Distract	0.255	0.002	–	–
Distract_7 -> Distract	0.205	0.029	–	–
IMotive_1 < - IMotive	–	–	0.737	<0.001
IMotive_1 < - Ethics	–	–	0.817	<0.001
IMotive_2 < - IMotive	–	–	0.801	<0.001
IMotive_2 < - Awareness	–	–	0.826	<0.001
IMotive_3 < - IMotive	–	–	0.813	<0.001
IMotive_3 < - Ethics	–	–	0.848	<0.001
IMotive_5 < - IMotive	–	–	0.535	<0.001
IMotive_5 < - Awareness	–	–	0.617	<0.001
IMotive_6 < - IMotive	–	–	0.674	<0.001
IMotive_6 < - Awareness	–	–	0.780	<0.001
IMotive_7 < - Ethics	–	–	0.575	<0.001
IMotive_7 < - IMotive	–	–	0.478	<0.001
EMotive_9 -> EMotive	0.639	<0.001	–	–
EMotive_11 -> EMotive	0.432	<0.001	–	–
SRPract_1 -> SRPract	0.265	<0.001	–	–
SRPract_2 -> SRPract	0.167	<0.001	–	–
SRPract_3 -> SRPract	0.227	<0.001	–	–
SRPract_4 -> SRPract	0.275	<0.001	–	–
SRPract_5 -> SRPract	0.273	<0.001	–	–
SRPract_6 -> SRPract	0.202	<0.001	–	–

*Outer weight = formative factor; Outer loading = reflective factor*.

Additionally, Table 4 presents factor loadings of reflective constructs within a matrix.

**Table 4:** Factor loading matrix for reflective factors.

	**CMotive**	**IMotive**	**PAttitude**	**PObject**	**SRPract**	**SelfEff**
IMotive_1	0.226	**0.737**	0.356	−0.152	−0.236	0.222
IMotive_2	0.185	**0.801**	**0.401**	−0.266	−0.294	0.274
IMotive_3	0.232	**0.813**	**0.439**	−0.232	−0.267	0.288
IMotive_5	0.129	**0.535**	0.294	−0.101	−0.192	0.23
IMotive_6	0.115	**0.674**	0.349	−0.24	−0.232	0.259
IMotive_7	0.143	**0.478**	0.252	−0.141	−0.151	0.27
SRPract_1	−0.123	−0.205	−0.121	0.318	**0.697**	−0.273
SRPract_2	0.062	−0.179	−0.094	0.268	**0.524**	−0.031
SRPract_3	−0.093	−0.243	−0.16	0.23	**0.74**	−0.201
SRPract_4	−0.126	−0.291	−0.256	0.242	**0.786**	−0.272
SRPract_5	−0.172	−0.247	−0.243	0.268	**0.774**	−0.247
SRPract_6	0.004	−0.263	−0.13	0.299	**0.653**	−0.183
PAttitude_2	0.202	**0.426**	**0.752**	−0.179	−0.239	0.289
PAttitude_4	0.243	0.351	**0.68**	−0.214	−0.149	0.285
PAttitude_5	0.201	**0.417**	**0.796**	−0.194	−0.219	0.28
PAttitude_6	0.168	0.192	**0.509**	−0.093	−0.028	0.261
PObject_1	−0.056	−0.228	−0.228	**0.797**	0.304	−0.178
PObject_2	−0.036	−0.217	−0.201	**0.768**	0.25	−0.092
PObject_3	−0.011	−0.219	−0.191	**0.718**	0.288	−0.071
PObject_4	−0.048	−0.131	−0.061	**0.593**	0.234	−0.168
PObject_5	−0.12	−0.199	−0.175	0.675	0.278	−0.14
SelfEff_1	0.145	0.238	0.265	−0.12	−0.226	**0.707**
SelfEff_2	0.187	0.362	0.399	−0.243	−0.269	**0.704**
SelfEff_3	0.239	0.245	0.28	−0.073	−0.197	**0.785**
SelfEff_4	0.233	0.231	0.203	−0.067	−0.188	**0.761**
CMotive_1	**0.874**	0.226	0.285	−0.066	−0.123	0.248
CMotive_2	**0.816**	0.172	0.194	−0.045	−0.081	0.259
CMotive_3	**0.9**	0.263	0.272	−0.077	−0.126	0.208
CMotive_4	**0.863**	0.222	0.254	−0.079	−0.098	0.214

Reflective constructs show that all of their items have adequate loadings, which is generally expected to be above 0.4 on their respective factor. On the other hand, some items also load above this threshold in different factors than the one expected. Specifically, PAttitude and IMotive show some of their items cross-load with each other: IMotive_2 and IMotive_3 with 0.401 and 0.439 in PAttidude; PAttitude_2 and PAttitude_5 with 0.426 and 0.417 in IMotive. This makes contextual sense, given both constructs deal with personal values regarding HCAI control practices, however, both are questioned differently and look for different aspects of these values (i.e., IMotive strictly asks the drive to comply, while PAttitude asks for their attitudes toward the risk of infection). Cross-loading items also present a considerable difference between each factor they load to (i.e., primary loading should be at least 0.2 larger than the secondary), suggesting these not necessarily merit deletion. Following this reasoning, the items were retained for the scale.

Twenty-one items presented coefficients with patterns that suggested a second-order structure. We identified a total of nine second-order factors across four main factors. Specifically, HEPractice shows a distinction between items about laboratory (e.g., student to teacher ratio and sufficient laboratory classes), and resources in practice-based courses (e.g., receiving feedback, technological resources, and simulation sessions). HICulture shows a distinction between items that relate to giving feedback in clinical placements (e.g., receiving guidance and correction) and having role models (e.g., others following example, getting motivated to comply with safe practices, and having supervisors ensure protective equipment is being used). Fatigue shows a distinction between items related to academic-related workload (e.g., clinical placements when tired and academic overload) and general fatigue (e.g., mental, physical, and emotional). IMotive shows a distinction between items about ethical guidelines (e.g., driven by ethics, patient health, and quality care) than from items that relate to awareness of what HCAI entails (e.g., aware of consequences, self-protection, and desire to prevent it). Emotive shows a distinction between items that reflect approval (e.g., meet expectations of supervisor and approval of peers) and the structure of another second-order factor with a packet of two items that did not share a common factor. The text in these two items was “I will get better grades,” and “Other teams also follow them.” These two rogue items make sense as external motives, but not as a single second-order, and were marked for deletion. Table 5 shows second-order text and the relationship to the parent factor.

**Table 5:** Second-order factors.

**Parent factor**	**Second order**	**Item**	**Text**
HEPractice			
	Laboratory	HEPractice_2	There is an adequate student-to-teacher ratio in laboratory classes.
	Laboratory	HEPractice_3	There are enough laboratory classes where I can develop my competencies.
	Resource	HEPractice_1	Students have adequate technological resources (e.g., manikins, medical/clinical devices) to develop their competencies.
	Resource	HEPractice_4	Simulation is used as a learning method for students to develop their competencies in a structured setting.
HICulture			
	Feedback	HICulture_4	I receive guidance from my supervisor on adequate HCAI prevention and control measures.
	Feedback	HICulture_6	If I do not follow HCAI prevention and control measures, I receive feedback to improve my performance.
	Compliance	HICulture_2	My colleagues follow the safety protocols and procedures for HCAI prevention and control.
	Compliance	HICulture_5	My supervisor makes sure that we use the personal protective equipment available.
	Compliance	HICulture_7	The nursing team motivates me to follow HCAI prevention and control measures.
Fatigue			
	Academic Fatigue	Fatigue_1	I have experienced academic overload.
	Academic Fatigue	Fatigue_3	Clinical placements have coincided with periods when I was feeling tired.
	General Fatigue	Fatigue_2	I have experienced difficult moments in my personal life.
	General Fatigue	Fatigue_4	I have felt emotionally tired.
	General Fatigue	Fatigue_5	I have felt mentally tired.
	General Fatigue	Fatigue_6	I have felt physically tired.
IMotive			
	Ethic	IMotive_1	My main drive is patient health.
	Ethic	IMotive_3	I aim for quality care.
	Ethic	IMotive_7	I am driven by professional ethics.
	Awareness	IMotive_2	I understand its implications.
	Awareness	IMotive_5	I am concerned about my own safety.
	Awareness	IMotive_6	I want to implement prevention measures.

Reliability and convergent validity results for reflective factors show adequate values: CMotivation (CR = 0.921; AVE = 0.746); SelfEff (CR = 0.828; AVE = 0.547); PObject (CR = 0.837; AVE = 0.510); SRPract (CR = 0.851; AVE = 0.492); PAttitude (CR = 0.783; AVE = 0.480); IMotive (CR = 0.837; AVE = 0.469). While some of the AVE values are below the cutoff value of 0.5, we believe that CR may suffice as it is, per the suggestions by Malhotra and Dash (2011).

According to the analysis, the VIF ranged from 1.3 to 4.3, the highest items being Fatigue_4 (4.042) and Fatigue_5 (4.317), with the rest of the items averaging around 1.7. Looking at the item text in both: “I have felt emotionally tired,” and “I have felt mentally tired,” it makes sense to obtain high VIF values, however, mental and emotional fatigue are logically distinct on their own. As they correspond to the same factor, we decided that their VIF is not a concern. No items were found to have collinearity issues, and none marked for deletion.

Lastly, after all these adjustments, SRMR showed 0.064 and 0.076 values for the saturated and estimated model, respectively, and represent a good model fit.

**Figure 2:**
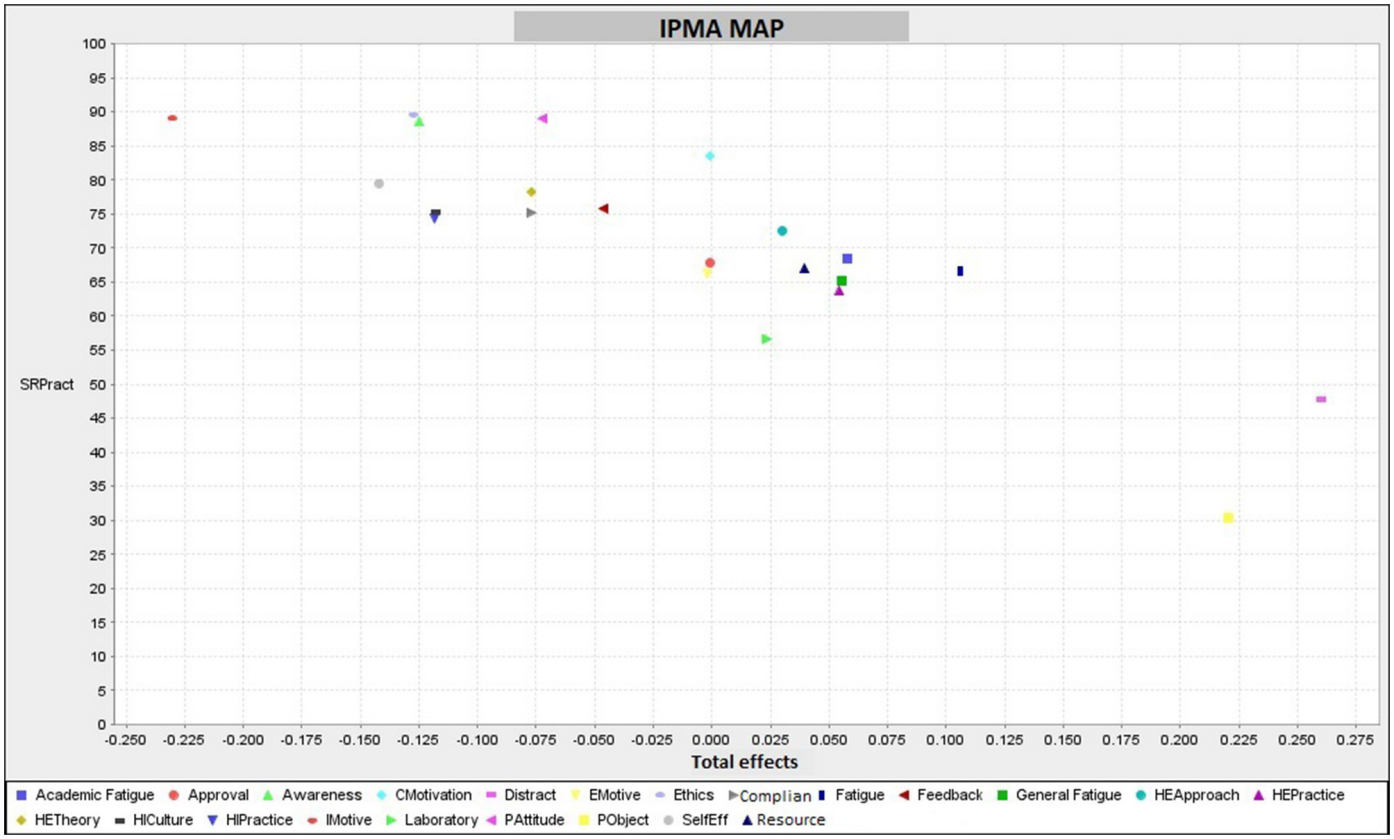
IPMA map.

Table 6 presents descriptive information of each factor (number of items, mean, SD, and kurtosis), and Figure 2 shows the Importance-Performance Map Analysis (IPMA) graph, which determines the relative importance of the constructs within the scale.

**Table 6:** Total items per factor, mean, and SD.

						**Skewness**		**Kurtosis**	
	**No. Items**	**Minimum**	**Maximum**	**Mean**	**STD**	**Statistic**	**Std. Error**	**Statistic**	**Std. Error**
HETheory	6	2.00	5.00	4.1410	0.54708	−0.600	0.095	0.404	0.191
HEApproach	5	1.60	5.00	3.8165	0.70745	−0.370	0.095	−0.180	0.191
HEPractice	4	1.00	5.00	3.4745	0.89112	−0.224	0.095	−0.487	0.191
HIPractice	6	1.67	5.00	3.9759	0.59860	−0.506	0.095	0.042	0.191
HICulture	6	1.83	5.00	4.0765	0.65288	−0.644	0.095	0.095	0.191
PAttitude	4	2.75	5.00	4.5114	0.45343	−1.063	0.095	0.982	0.191
PObject	5	1.00	5.00	2.2482	0.76902	0.683	0.095	0.799	0.191
SelfEff	4	2.25	5.00	4.1391	0.56672	−0.603	0.095	0.439	0.191
CMotive	4	1.25	5.00	4.3365	0.75060	−1.335	0.095	1.722	0.191
Fatigue	6	1.00	5.00	3.6662	0.85778	−0.383	0.095	−0.354	0.191
Distract	5	1.00	5.00	2.9302	0.71916	−0.066	0.095	0.134	0.191
IMotive	6	2.17	5.00	4.5998	0.40874	−1.478	0.095	3.456	0.191
Emotive	2	1.00	5.00	3.7393	1.05854	−0.837	0.095	0.214	0.191
SRPract	6	1.00	5.00	1.7594	0.67589	1.456	0.095	3.091	0.191

## Discussion

### Description of the Final Version of the Questionnaire

The development process of the InovSafeCare scale started with the question: “what factors within this framework comprise the student HCAI control and prevention ecosystem?” After a process of qualitative item development, and empirical testing through a quantitative study to answer this question, the resulting scale led to 81 concise items representing the following 14 factors: Higher Education Institution HCAI Theoretical Content, Higher Education Institution Approach to teaching HCAI, Higher Education Institution HCAI practice-related approach/resources, Health Institution HCAI prevention practices, Health Institution HCAI prevention culture, Personal HCAI Attitudes, Personal objection to HCAI prevention and control measures, Self-Efficacy on HCAI prevention and control, Career Motivation, Fatigue, Distract, Intrinsic motives to comply with HCAI control and prevention measures, Extrinsic motives to comply with HCAI control and prevention measures, and Self-reported HCAI practices. These studies evidenced the validity of the construct and adequate psychometric properties of the factors presented.

The constructs, item, and text are presented in Table 7.

**Table 7:** InovSafeCare Scale (81 items).

**Higher Education Institution HCAI Theoretical Content**	
1- Completely Disagree; 2- Partially Disagree; 3- Neither Agree nor Disagree; 4- Partially Agree; 5- Completely Agree	
	*Are the following Healthcare-Associated Infection (HCAI) topics addressed in your Educational Institution?*
HETheory_3	Hand hygiene.
HETheory_5	Use of personal protective equipment
HETheory_6	Decontamination of medical/clinical devices and equipment
HETheory_9	Proper clinical waste management
HETheory_11	Prevention of exposure to microbial agents in the workplace
HETheory_12	Additional precautions for specific transmission routes.
**Higher Education Institution Approach to teach HCAI**	
1- Completely Disagree; 2- Partially Disagree; 3- Neither Agree nor Disagree; 4- Partially Agree; 5- Completely Agree	
	*How is the content on HCAI prevention and control delivered in your Educational Institution?*
HEApproach_1	There are mandatory courses dedicated to the development of competencies.
HEApproach_2	HCAI prevention and control topics are included in theoretical classes.
HEApproach_3	Students are offered workshops or classes by professionals.
HEApproach_4	Visual resources and other technologies are used in classrooms to explain HCAI topics.
HEApproach_5	My teachers discuss HCAI prevention and control measures on class subjects when relevant.
**Higher Education Institution HCAI practice-related approach/resources**	
1- Completely Disagree; 2- Partially Disagree; 3- Neither Agree nor Disagree; 4- Partially Agree; 5- Completely Agree	
	*How are the practical contents on HCAI prevention and control taught at your Educational Institution?*
HEPractice_1	Students have adequate technological resources (e.g., manikins, medical/clinical devices) to develop their competencies.
HEPractice_2	There is an adequate student-to-teacher ratio in laboratory classes.
HEPractice_3	There are enough laboratory classes where I can develop my competencies.
HEPractice_4	Simulation is used as a learning method for students to develop their competencies in a structured setting.
**Health Institution HCAI prevention practices**	
1- Never; 2- Rarely; 3- Sometimes; 4- Often; 5- Always	
	*What are the HCAI prevention and control measures in the Health Institutions where you do your clinical placements?*
HIPractice_1	Hand hygiene supplies are available in each patient and treatment room.
HIPractice_2	There is a “Bare below the Elbows” policy (e.g., wristwatches, rings, and bracelets should not be worn).
HIPractice_3	Specialized/infection control link nurses are involved in my clinical rotations.
HIPractice_4	HCAI-related resources are accessible to all professionals (e.g., equipment, protocols and regulations).
HIPractice_5	There are visual reminders in key areas to improve hand hygiene compliance (e.g., corridors, treatment rooms).
HIPractice_7	Personal protective equipment is available in key areas (e.g., outside the isolation rooms).
**Health Institution HCAI prevention culture**	
1- Never; 2- Rarely; 3- Sometimes; 4- Often; 5- Always	
	*What is the HCAI prevention and control culture in the Health Institution where you do your clinical placements?*
HICulture_1	Hand hygiene is promoted in the clinical area.
HICulture_2	My colleagues follow the safety protocols and procedures for HCAI prevention and control.
HICulture_4	I receive guidance from my supervisor on adequate HCAI prevention and control measures.
HICulture_5	My supervisor makes sure that we use the personal protective equipment available.
HICulture_6	If I do not follow HCAI prevention and control measures, I receive feedback to improve my performance.
HICulture_7	The nursing team motivates me to follow HCAI prevention and control measures.
**Personal HCAI Attitudes**	
1- Completely Disagree; 2- Partially Disagree; 3- Neither Agree nor Disagree; 4- Partially Agree; 5- Completely Agree	
	*What are your attitudes toward the risks of infection in your Health Institutions?*
Pattitude_2	As a student, I feel responsible for preventing and controlling the spread of infections.
Pattitude_4	I believe that all protocols for HCAI prevention and control must be followed.
Pattitude_5	I believe that my safety measures contribute to preventing the spread of infections.
Pattitude_6	I value theoretical recommendations more than my own opinion.
**Personal objection to HCAI prevention and control measures**	
1 - Not at all; 2 - Rarely; 3 - Sometimes; 4 - Often; 5 - Always	
	*During your clinical placements, did you have any objections to HCAI prevention and control measures?*
Pobject_1	Some HCAI safety protocols are unnecessary.
Pobject_2	I express my reservations to the application of HCAI prevention and control measures that I deem unnecessary.
Pobject_3	The use of so much disposable material is unnecessary.
Pobject_4	Personal protective equipment is uncomfortable.
Pobject_5	I feel upset if my supervisor is constantly monitoring my HCAI prevention and control measures.
**Self-efficacy on HCAI prevention and control**	
1- Completely Disagree; 2- Partially Disagree; 3- Neither Agree nor Disagree; 4- Partially Agree; 5- Completely Agree	
	*How confident do you feel about your ability to follow the safety measures for HCAI prevention and control?*
SelfEff_1	I find it easy to comply with the safety protocols.
SelfEff_2	Even if others did not, I would still follow the safety measures.
SelfEff_3^*^	I am confident that I could deal efficiently with HCAI prevention and control measures under unexpected events (e.g., unexpected patient transfer).
SelfEff_4^*^	I am confident that I could deal efficiently with HCAI prevention and control measures under stressful events (e.g., performing a nursing procedure for the first time).
**Career Motivation**	
1- Completely Disagree; 2- Partially Disagree; 3- Neither Agree nor Disagree; 4- Partially Agree; 5- Completely Agree	
	*How motivated do you feel about your future as a nurse?*
Cmotivation_1	I am satisfied with my career choice.
Cmotivation_2	My clinical placements keep me motivated into pursuing a career as a nurse.
Cmotivation_3	I am very interested in continuing a career as a nurse.
Cmotivation_4	Being a nurse will fulfill my professional expectations.
**Fatigue**	
1- Never; 2- Rarely; 3- Sometimes; 4- Often; 5- Always	
	*To what extent have the following applied to you during the past 4 months?*
Fatigue_1	I have experienced academic overload.
Fatigue_2	I have experienced difficult moments in my personal life.
Fatigue_3	Clinical placements have coincided with periods when I was feeling tired.
Fatigue_4	I have felt emotionally tired.
Fatigue_5	I have felt mentally tired.
Fatigue_6	I have felt physically tired.
**Distraction**	
1- Never; 2- Rarely; 3- Sometimes; 4- Often; 5- Always	
	*During your clinical placements, do you get distracted and/or lose focus on your tasks because of:*
Distract _1	Disorganized rooms or units.
Distract _2	Too many patients in the same room/space.
Distract _5	Caring for patients with multiple conditions.
Distract _6	Emotionally demanding cases.
Distract _7	Having to perform multiple clinical tasks.
**Intrinsic motives to comply with HCAI control and prevention measures**	
1- Completely Disagree; 2- Partially Disagree; 3- Neither Agree nor Disagree; 4- Partially Agree; 5- Completely Agree	
	*What drives you to follow HCAI prevention and control procedures?*
Imotive_1	My main drive is patient health.
Imotive_2	I understand its implications.
Imotive_3	I aim for quality care.
Imotive_5	I am concerned about my own safety.
Imotive_6	I want to implement prevention measures.
Imotive_7	I am driven by professional ethics.
**Extrinsic motives to comply with HCAI control and prevention measures**	
1- Completely Disagree; 2- Partially Disagree; 3- Neither Agree nor Disagree; 4- Partially Agree; 5- Completely Agree	
	*What drives you to follow HCAI prevention and control procedures?*
Emotive_2	Other team members would think better of me.
Emotive_4	I will meet the expectations of my supervisor.
**Self-reported HCAI practices**	
1- Completely Disagree; 2- Partially Disagree; 3- Neither Agree nor Disagree; 4- Partially Agree; 5- Completely Agree	
	*During your clinical placements, have any of the following situations occurred?*
SRPract_1	I have forgotten to follow safety protocols when required.
SRPract_2	I have worn accessories below the elbows (e.g., wrist watches, bracelets, rings,).
SRPract_3	I have not used gloves when required.
SRPract_4	I have not washed and/or disinfected my hands when required.
SRPract_5	I have not worn face masks when required.
SRPract_6	I have assisted a patient without changing my gloves, masks or other protective equipment.

* *SelfEff_3 and SelfEff_4 are items adapted from the General Self Efficacy (GSE) Scale by Schwarzer and Jerusalem (1995)*.

### Use and Future Research

The development of this scale serves both, a research-focused purpose to answer the question “How does the HCAI prevention and control practices ecosystem for students interact within it?”, and a practical purpose, which would be to answer the question “what can we do within this ecosystem to improve safe practices of students?” We use the term ecosystem as an umbrella term in this study to start to thread a theoretical framework, and this scale is a step further to understand, with a more empirical approach, where and how we should focus on improving the quality and practicality of education, work culture, and attitudes in nurse students to lead to a more efficient handling of HCAI breaches during clinical placements, and potential future as a full practitioner.

Future research should start with developing a theoretical model of how these variables interact within themselves and their relevance. A more robust approach would be to use this information to create new training modules that can help improve the ecosystem and use a longitudinal approach to see how it improves real-life performance metrics (e.g., HCAI cases) with its implementation.

### Limitations

The scale utilizes self-reported measures, meaning there is always respondent bias, and its length is also prone to respondent fatigue. Second, method-wise, PLS-SEM is a recent approach to measure and validate formative constructs and structural models, and while this is a current challenge to research into these type of constructs (Hair et al., 2018), we nonetheless used the most current accepted methods to provide the best indicators of validity. On the other hand, although PLS-SEM does not rely on the conventional 1:10 rule of thumb for sample adequacy, the sample may benefit from a bigger pool of participants, as well as a concurrent validity analysis in a future study. Third, the sample represents members of a European consortium of four countries (which itself is a sample of the European Union (EU) population) but would have benefited from more countries within the zone participating. To compensate for this limitation, the foundations behind the questionnaire were guided by the Bologna Declaration 1999's plan in higher education, which provides a uniform guideline for safe practices for all EU members.

## Data Availability Statement

The datasets presented in this article are not readily available because the dataset remains for exclusive use by the authors due to participant privacy and informed consent. Requests to access the datasets should be directed to Amaia Yurrebaso Macho, amaiay@usal.es.

## Ethics Statement

The studies involving human participants were reviewed and approved by Ethics Committees (Health Sciences Research Unit: Nursing (UICISA: E) of the Nursing School of Coimbra—reference 635/11-2019). The patients/participants provided their written informed consent to participate in this study.

## Author Contributions

All authors listed have made a substantial, direct, and intellectual contribution to the work, and approved it for publication.

## Funding

This study was part of the InovSafeCare Project, Educating Students for innovative infection prevention and control practices in healthcare settings (2018-1-PT01-KA203-047453), co-funded by the Erasmus+ Programme of the European Union with the support of the c (Portugal).

## Conflict of Interest

All authors declare that the research was conducted in the absence of any commercial or financial relationships that could be construed as a potential conflict of interest.

## Publisher's Note

All claims expressed in this article are solely those of the authors and do not necessarily represent those of their affiliated organizations, or those of the publisher, the editors and the reviewers. Any product that may be evaluated in this article, or claim that may be made by its manufacturer, is not guaranteed or endorsed by the publisher.
